# Clinical determination of anatomical diameter in different dental groups correlating them with gender, age, tooth/canal and pulpoperiradicular diagnosis: an observational clinical study

**DOI:** 10.1038/s41598-023-41967-9

**Published:** 2023-09-27

**Authors:** Ricardo Machado, Gabriel Filipe Pamplona, Claudemir de Souza Júnior, Jaqueline Nascimento, Eduardo Donato Eing Elgelke Back, Daniel Comparin, Sérgio Aparecido Ignácio, Stella Maria Glaci Reinke, Ana Cristina Kovalik, Ulisses Xavier da Silva Neto

**Affiliations:** 1 Department of Endodontics, School of Dentistry, Regional University of Blumenau-FURB, Rua São Paulo, 2171, Itoupava Seca, Blumenau, Santa Catarina 89030-001 Brazil; 2 Private Clinic, Foz do Iguaçú, Paraná Brazil; 3 Department of Endodontics, School of Dentistry, Pontifical Catholic University of Paraná-PUC/PR, Curitiba, Paraná Brazil; 4 Private Clinic, Joinville, Santa Catarina Brazil; 5 Department of Endodontics, School of Dentistry, Paranaense University-UNIPAR, Francisco Beltrão, Paraná Brazil; 6 Department of Biostatistics, School of Dentistry, Pontifical Catholic University of Paraná-PUC/PR, Curitiba, Paraná Brazil; 7 Department of Periodontics, School of Dentistry, Regional University of Blumenau-FURB, Blumenau, Santa Catarina Brazil

**Keywords:** Health care, Dentistry, Endodontics

## Abstract

The aim of this observational clinical study (OCS) was to determine the clinical anatomical diameter (CAD) in several dental groups, thus correlating them with gender, age, tooth/canal and pulpoperiradicular diagnosis. Three-hundred fifty-nine teeth/584 vital or necrotic root canals from patients of both genders and different ages composed the sample. After performing the necessary previous procedures, K-Flexofiles were used to determine the CAD. Then, the gender and age of the patients, as well as the pulpoperiradicular diagnosis of the teeth were tabulated to conduct the statistical analysis (p < 0.05). Of the 359 teeth/584 root canals evaluated, 208/342 were from women (mean age 38.85 ± 13.42 years) and 151/242 were from men (mean age 45.41 ± 14.90 years). Statistically significant differences between the CAD means of root canals from women and men were not identified (p = 0.411). The analysis of the correlation between the CAD and age also showed a total independence (p = 0.271). Teeth with pulp necrosis and asymptomatic apical periodontitis diagnosed radiographically (TPNAAPDR) had a significantly larger CAD mean than teeth with pulp necrosis and no asymptomatic apical periodontitis diagnosed radiographically (TPNNAAPDR) and teeth with vital pulp and normal apical tissues (TVPNAT) (p = 0.0297); and the last two did not differ statistically (p > 0.05). The largest CAD mean was observed in single canals of maxillary central incisors. The lowest values of this variable were identified in the mesiobuccal and mesial canals of maxillary and mandibular first molars, respectively. The CAD of the root canals was influenced only by the root canal/tooth and pulpoperiradicular diagnosis.

## Introduction

The main purpose of endodontic treatment is to maintain or restore the health of periapical tissues. In vital teeth, the canal is emptied, cleaned, shaped, and hermetically filled with a biocompatible material. Due to the non-compromising of the periradicular tissues, the treatment aims to keep their integrity. On the other hand, a necrotic pulp is no longer able to prevent microbial invasion, thus allowing the establishment of endodontic infection, followed by the development of apical periodontitis. Therefore, in these cases, the treatment aims the restoration of tissue normality through cleaning and disinfection of the root canal system (RCS) obtained, above all, by biomechanical preparation, which are later preserved by the filling and coronal sealing^1^.

An efficient biomechanical preparation is only achieved through determining a correct apical limit, associated with an adequate instrumentation amplitude^2^. The most important studies carried out to evaluate the prognosis of endodontic treatment reported considerable success rates from the institution of the apical instrumentation limit near the cement-dentin-canal junction^3–7^. Therefore, despite the existence of different philosophical trends^8–10^, scientific evidence recommends the working length is set at 0.5–1.0 mm from the major apical foramina^11,12^.

On the other hand, the amplitude of apical instrumentation is a controversial subject in Endodontics^13,14^. Minor apical dilatations have been recommended to enhance the longevity of endodontically treated teeth in the oral cavity^15^; however, they can affect the prognosis of endodontic treatment due to the impairment of the cleaning and disinfection process, represented by the greater amount of root canal walls that were not “covered” by the instrumentation^16^.

However, the clinical anatomical diameter (CAD) of the root canal must be determined^17–19^, regardless of the subsequent implementation of greater or lesser amplitude of apical instrumentation^14^. Despite the advent of new proposals based on the use of imaging exams and resources^20,21^, the most used method for this purpose recommends the use of fine-caliber endodontic instruments in ascending order of diameter, until the identification of the one that best fits the root canal walls at the working length. This way it is possible to determine the CAD^17–19^.

To date, almost all scientific investigations carried out to determine the anatomical diameters of the root canals in different dental groups have been conducted in vitro^17–19,21–23^. Even recognizing the important information obtained by these researches, the impossibility of analyzing the likely impacts caused by “extrinsic factors”, such as gender, age, tooth/canal and pulpoperiradicular diagnosis, for example, on the dimensions of the pulp cavity, needs to be taken into account.

The objective of this OCS was to determine the CAD in different dental groups correlating them with gender, age, tooth/canal and pulpoperiradicular diagnosis. As a null hypothesis, it was considered that the CAD is not influenced by the abovementioned factors.

## Materials and methods

### Approval by the research ethics committee

This study was approved by the Research Ethics Committee of the Paranaense University—UNIPAR, Campus Francisco Beltrão, Paraná, Brazil (CAAE: 45486015.2.0000.0109) on 05/28/2015, is registered at the Brazilian Registry of Clinical Trials (RBR-7wfrs6k), and it was performed considering the principles of the World Medical Association Declaration of Helsinki “Ethical Principles for Medical Research Involving Human Subjects”, (amended in October 2013). Informed consent was obtained from each patient or guardians of patients under 18 years of age, who participated in the study.

### Sample selection

The sample of this OCS consisted of patients with complete rhizogenesis teeth, who had indications to undergo endodontic treatment for different reasons, who were attended during the clinical activities of the Disciplines of Multidisciplinary Clinic I and II and Integrated Clinic Internship I and II of the School of Dentistry, Paranaense University—UNIPAR, Campus Francisco Beltrão, Paraná, Brazil, between May 2015 and July 2016. Such indications were confirmed through anamnesis, clinical examination (inspection, palpation, percussion, mobility, and periodontal probing), cold pulp thermal test using refrigerant gas (EndoIce; Coltene/Whaledent Inc., Cuyahoga Falls, Ohio, United States) and radiographic examination using the parallelism technique. Previously treated teeth and/or affected by abrupt curvatures (between 20° and 75°)^24^, root resorptions or other relevant anatomical features (severe atresia, presence of nodules or needle calcifications, etc.) were promptly excluded. Based on the information provided by the aforementioned exams and tests, the teeth were classified as teeth with vital pulp and normal apical tissues (TVPNAT), teeth with pulp necrosis and no asymptomatic apical periodontitis diagnosed radiographically (TPNNAAPDR) and teeth with pulp necrosis and asymptomatic apical periodontitis diagnosed radiographically (TPNAAPDR). For each tooth, the diagnosis was confirmed after endodontic access. After analyzing clinical-radiographic evidence of diagnostic duality (asymptomatic apical periodontitis present only in one or two roots in multirooted teeth, vital and necrotic canals in the same tooth, etc.), we opted for exclusion. Summarizing, a single pulpoperiradicular diagnosis was established for each tooth, regardless of the number of roots and canals.

### Clinical procedures

After anesthesia (2% lidocaine and 1:80.000 epinephrine—DFL, Rio de Janeiro, Rio de Janeiro, Brazil) and placement of the rubber dam, endodontic access was performed with spherical drills no. 1014HL or 1016HL and conical burs with inactive tip. no. 3082, 3083 or Endo Z (Dentsply-Maillefer, Ballaigues, Switzerland), according to the coronal volume. Then, the orifice entrances and the cervical and middle thirds of the root canals were, respectively, prepared with Largo and Gates–Glidden drills no. I, II, III or IV (Dentsply-Maillefer), based on information obtained through radiographic examination and initial exploration, previously performed with a no. 10 or 15 K-File (Dentsply-Maillefer). Sodium hypochlorite (NaOCl) was used as irrigant solution at 2.5 and 5.25% (Fórmula & Ação, São Paulo, São Paulo, Brazil) for vital and necrotic teeth, respectively, by means of a NaviTip needle (Ultradent, South Jordan, Utah, USA), initially calibrated at 5 mm from the radiographic apex. Then, a no. 08, 10, 15 or 20 K-File (Dentsply-Maillefer), connected to an electronic apex locator (Novapex; Forum, Israel), was used to obtain apical patency, later confirmed by radiographic examination, thus establishing the working length near the apical constriction by subtracting 1 mm from the apical foramen (0.0 mm). Subsequently, K-Flexofiles (from no. 08) were used in increasing diameter order. For each root canal, CAD was determined by the instrument that best fit the root canal walls at the working length (around the apical constriction). The patients' gender and age, as well as the pulpoperiradicular diagnosis and CAD were tabulated in an Excel spreadsheet (Microsoft Corporation, Redmond, Washington, USA) for statistical analysis.

### Statistical analysis

Considering the normality of the data was confirmed by means of the Kolmogorov–Smirnov test, the following tests were applied for the other assessments: parametric Student's *t*, Pearson's parametric correlation, One-way analysis of variance for independent samples, Levene, non-parametric Kruskal-Wallis and Games-Howell multiple parametric comparisons for heterogeneous variances (p < 0.05). Statistical analysis was performed using SPSS 25.0 software (IBM Corp., Armonk, NY, United States)^25^.

## Results

A total of 359 teeth/584 root canals from patients with a mean age of 41.61 ± 14.41 years were evaluated in the present study – 208 teeth/342 canals from female patients (mean age 38.85 ± 13.42 years) and 151 teeth/242 canals from male patients (mean age 45.41 ± 14.90 years). The most frequently treated teeth were vital or necrotic (with or without asymptomatic apical periodontitis diagnosed radiographically) maxillary premolars and vital maxillary first molars (Table 1).

**Table 1:** Tooth, number and percentage of root canals according to gender and pulpoperiradicular diagnosis.

Tooth	n./% of canals (male patients)			n./% of canals (female patients)			n./% of canals (total)		
	TVPNAT*	TPNNAAPDR**	TPNAAPDR***	TVPNAT*	TPNNAAPDR**	TPNAAPDR***	TVPNAT*	TPNNAAPDR**	TPNAAPDR***
**Maxillary central incisor**	7/1.95%	2/0.56%	12/3.34%	2/0.56%	3/0.84%	20/5.57%	9/2.51%	5/1.39%	**32/8.91%**
Maxillary lateral incisor	1/0.28%	1/0.28%	10/2.79%	3/0.84%	2/0.56%	14/3.9%	4/1.11%	3/0.84%	24/6.69%
Maxillary canine	3/0.84%	3/0.84%	6/1.67%	2/0.56%	2/0.56%	7/1.95%	5/1.39%	5/1.39%	13/3.62%
**Maxillary first premolar**	6/1.67%	2/0.56%	12/3.34%	7/1.95%	5/1.39%	16/4.46%	**13/3.62%**	**7/1.95%**	**28/7.8%**
**Maxillary second premolar**	5/1.39%	2/0.56%	9/2.51%	9/2.51%	6/1.67%	18/5.01%	**14/3.9%**	**8/2.23%**	**27/7.52%**
**Maxillary first molar**	2/0.56%	3/0.84%	5/1.39%	10/2.79%	2/0.56%	3/0.84%	**12/3.34%**	5/1.39%	8/2.23%
Maxillary second molar	2/0.56%	2/0.56%	1/0.28%	2/0.56%	0/0%	2/0.56%	4/1.11%	2/0.56%	3/0.84%
Maxillary third molar	0/0%	0/0%	0/0%	0/0%	0/0%	0/0%	0/0%	0/0%	0/0%
Mandibular central incisor	0/0%	0/0%	6/1.67%	0/0%	0/0%	3/0.84%	0/0%	0/0%	9/2.51%
Mandibular lateral incisor	2/0.56%	1/0.28%	5/1.39%	2/0.56%	1/0.28%	6/1.67%	4/1.11%	2/0.56%	11/3.06%
Mandibular canine	0/0%	1/0.28%	4/1.11%	0/0%	2/0.56%	3/0.84%	0/0%	3/0.84%	7/1.95%
Mandibular first premolar	1/0.28%	2/0.56%	4/1.11%	2/0.56%	3/0.84%	6/1.67%	3/0.84%	5/1.39%	10/2.79%
Mandibular second premolar	4/1.11%	1/0.28%	9/2.51%	5/1.39%	4/1.11%	13/3.62%	9/2.51%	5/1.39%	22/6.13%
Mandibular first molar	3/0.84%	1/0.28%	4/1.11%	5/1.39%	1/0.28%	5/1.39%	8/2.23%	2/0.56%	9/2.51%
Mandibular second molar	3/0.84%	2/0.56%	2/0.56%	2/0.56%	2/0.56%	8/2.23%	5/1.39%	4/1.11%	10/2.79%
Mandibular third molar	0/0%	0/0%	0/0%	0/0%	0/0%	0/0%	0/0%	0/0%	0/0%

Bold: Most frequently treated teeth.

*Teeth with vital pulp and normal apical tissues.

**Teeth with pulp necrosis and no asymptomatic apical periodontitis diagnosed radiographically.

***Teeth with pulp necrosis and asymptomatic apical periodontitis diagnosed radiographically.

No statistically significant differences were identified between the CAD means of the root canals of teeth from female (23.73 ± 8.49 × 10^−2^ mm) and male (23.14 ± 8.51 × 10^−2^ mm) patients (p = 0.411) (Table 2).

**Table 2:** Gender, number of evaluated canals, statistical data on CAD and p value.

Gender	n	CAD (× 10^−2^ mm)					p value**
		Mean*	Standard deviation	Standard error	Confidence interval (95%)		
					LI	LS	
Female	342	23.73^a^	8.497	0.459	22.82	24.63	0.411
Male	242	23.14^a^	8.512	0.547	22.06	24.22	

*Same letters indicate no statistically significant difference.

**Value obtained through Student's t test for independent samples.

The analysis of the correlation between the CAD and age showed the complete independence between them: r = − 0.046/p = 0.271 (general), r = − 0.009/p = 0.867 (female patients), and r = − 0.077/p = 0.233 (male patients) (Table 3).

**Table 3:** Statistical data referring to Pearson's correlation analysis between the CAD and age.

CAD	Pearson correlation	Age
General	Pearson correlation	− 0.046
	p value	0.271
	n	584
Female patients	Pearson correlation	− 0.009
	p value	0.867
	n	342
Male patients	Pearson correlation	− 0.077
	p value	0.233
	n	242

TPNAAPDR had a significantly larger CAD mean (24.88 ± 8.90 × 10^−2^ mm) than TPNNAAPDR (22.24 ± 8.17 × 10^−2^ mm) and TVPNAT (21.55 ± 7.38 × 10^−2^ mm) (p = 0.0297), and the last two did not differ statistically from each other (p > 0.05) (Table 4).

**Table 4:** Pulpal and periradicular diagnosis, number of evaluated canals, statistical data on the CAD, and p value.

Pulpal and periradicular diagnosis	n	CAD (× 10^−2^ mm)					p value^##^
		Mean^#^	Standard deviation	Standard error	Confidence interval (95%)		
					LI	LS	
TPNAAPDR*	320	24.88^a^	8.905	0.498	23.90	25.85	0.0001
TPNNAAPDR**	96	22.24^b^	8.172	0.834	20.58	23.90	
TVPNAT***	168	21.55^b^	7.381	0.569	20.42	22.67	

^#^Same letters indicate no statistically significant difference.

^##^Value obtained through the Games–Howell test (p < 0.05).

*Teeth with pulp necrosis and asymptomatic apical periodontitis diagnosed radiographically.

**Teeth with pulp necrosis and no asymptomatic apical periodontitis diagnosed radiographically.

***Teeth with vital pulp and normal apical tissues.

The largest CAD mean (38.26 ± 6.68 × 10^−2^ mm) was observed in single canals of maxillary central incisors. The lowest values of this variable were identified in the mesiobuccal canals of maxillary (17.00 ± 4.56 × 10^−2^ mm) and mandibular (17.37 ± 4.20 × 10^−2^ mm) first molars, and mesiolingual canals of mandibular first molars (15.79 ± 3.44 × 10^−2^ mm) (Table 5).

**Table 5:** Evaluated canals, quantity and statistical data referring to the CAD.

Canal/Tooth	n	CAD (× 10^−2^ mm)				
		Mean	Standard deviation	Standard error	Confidence interval (95%)	
					LI	LS
Single/Maxillary central incisor*	**46**	**38.26**	**6.685**	**0.986**	**36.28**	**40.25**
Single/Maxillary lateral incisor	31	26.94	5.428	0.975	24.94	28.93
Single/Maxillary canine	23	29.35	7.584	1.581	26.07	32.63
Buccal/Maxillary first premolar	48	18.85	4.864	0.702	17.44	20.27
Palatine/Maxillary first premolar	48	19.69	4.303	0.621	18.44	20.94
Buccal/Maxillary second premolar	33	21.36	6.763	1.177	18.97	23.76
Palatine/Maxillary second premolar	33	21.36	6.284	1.094	19.14	23.59
Single/Maxillary second premolar	16	26.25	9.220	2.305	21.34	31.16
Mesiobuccal/Maxillary first molar**	**25**	**17.00**	**4.564**	**0.913**	**15.12**	**18.88**
Distobuccal/Maxillary first molar	25	18.80	7.676	1.535	15.63	21.97
Palatine/Maxillary first molar	25	26.80	6.595	1.319	24.08	29.52
Mesiobuccal/Maxillary second molar	9	18.33	5.000	1.667	14.49	22.18
Distobuccal/Maxillary second molar	9	19.44	5.270	1.757	15.39	23.50
Palatine/Maxillary second molar	9	27.78	7.546	2.515	21.98	33.58
Single/Mandibular central incisor	9	23.33	6.614	2.205	18.25	28.42
Single/Mandibular lateral incisor	17	18.53	4.926	1.195	16.00	21.06
Single/Mandibular canine	10	30.00	10.274	3.249	22.65	37.35
Single/Mandibular first premolar	18	20.83	4.618	1.088	18.54	23.13
Single/Mandibular second premolar	36	27.50	6.381	1.063	25.34	29.66
Mesiobuccal/Mandibular first molar**	**19**	**17.37**	**4.206**	**0.965**	**15.34**	**19.40**
Mesiolingual/Mandibular first molar**	**19**	**15.79**	**3.441**	**0.789**	**14.13**	**17.45**
Distal/Mandibular first molar	19	26.32	5.973	1.370	23.44	29.19
Mesiobuccal/Mandibular second molar	19	18.68	4.360	1.000	16.58	20.79
Mesiolingual/Mandibular second molar	19	18.16	3.804	0.873	16.32	19.99
Distal/Mandibular second molar	19	31.05	9.216	2.114	26.61	35.49

*The largest CAD mean.

**The lowest CAD means.

## Discussion

A correct amplitude of instrumentation—essential for an adequate cleaning and disinfection process—is just achieved by establishing the initial volume or CAD of the root canal^17–19^. Up to date, the only clinical research related to the subject was carried out with the purpose of determining the CAD of the root canals of different dental groups comparing conventional K-Files and nickel-titanium rotary instruments without taper (Lightspeed). However, “extrinsic factors”, such as gender, age and pulpoperiapical diagnosis, were not taken into account^26^. Therefore, this is the first OCS specifically planned to determine and compare the CAD of the root canals from different dental groups, also investigating the potential effects of gender, age and pulpoperiapical diagnosis on this main variable. The sample consisted of 584 canals from 359 TVPNAT, TPNNAAPDR or TPNAAPDR—342 canals/208 teeth from female patients and 242 canals/151 teeth from male patients—with mean ages of 38.85 ± 13.42 and 45.41 ± 14.90 years, respectively. The null hypothesis was rejected because the CAD was influenced by the canal/tooth and pulpoperiradicular diagnosis.

For each canal, the CAD was determined by using K-Flexofiles in increasing diameter order until the identification of the instrument that best fit the root canal walls at the working length (around the apical constriction). This methodology has already been questioned, mainly due to the complexity of the root canal anatomy^19,20^. Furthermore, advanced imaging resources, such as cone-beam computed tomography (CBCT) and microcomputed tomography (micro-CT), may better determine the root canal dimensions^20^. However, despite the evolution of CBCT imaging, specific spatial measurements in the final millimeters of the root canal, such as the anatomical diameter, may be influenced by several factors, such as devices and softwares and the operator's experience and skills to manage the images provided by them. These factors may act as sources of bias, making this kind of measurement unreliable^27^. For these and other reasons, CBCT has not been clinically recommended for establishing the CAD in the guidelines published until the moment^28–30^.

Micro-CT is a much more accurate method to study different parameters related to the root canal anatomy^27^. However, it cannot be directly applied clinically^27,31^. As it is used only in laboratorial settings, it is impossible to evaluate potential correlations among the root canal anatomy and "extrinsic factors" as we did herein, once this type of research is carried out using extracted teeth or cadavers^27^. Then, despite its limitations, the only possible clinical method to determine the CAD correlating it with “extrinsic factors” is using endodontic files as we did herein. Machado et al.^32^, conducted a similar investigation; however, the main variable studied was obtaining apical or foraminal patency.

Permanent dentin production and deposition are the most responsible factors for changing the morphology of the pulp cavity, including the constriction of the root canals over time^33–36^. Forensic studies have been carried out comparing different methods for determining age estimates, among which the analysis of the dimensions of the pulp cavity through imaging exams and softwares^37–42^. While some studies have demonstrated the absence of significant correlations between the characteristics of internal dental anatomy in patients of both genders^37–40^, others have concluded that changes in pulp cavity volume were more evident in women^41,42^. The presence of specific estrogen receptors in human odontoblasts^43,44^, and the substrate synthesis of these cells from the lack of the aforementioned hormone in ovariectomized rats^45^, suggest its influence on odontoblastic performance, thus interfering with formation of secondary dentin and in the reduction of the space occupied by the pulp^42^. However, the results of the present scientific investigation demonstrated that there were no significant correlations between the CAD, gender and age. The concentration of patients in a relatively limited age group, that occurred randomly due to the nature of this clinical research, certainly contributed to this outcome. Furthermore, large multicenter studies carried out in vivo to analyze the morphology and internal dental anatomy highlighted the methodological particularities, size, and characteristics of the studied population (emphasizing the importance of the ethnic factor), as the main responsible factor for the dissimilarity of the results found, considering in association other demographic factors^46–48^. In other words, variables such as age and gender may or may not be relevant depending on the methodological and sampling characteristics of each study.

Canals of TPNAAPDR had the CAD mean significantly larger (24.88 ± 8.90 × 10^−2^ mm) than those of TPNNAAPDR (22.24 ± 8.17 × 10^−2^ mm) and TVPNAT (21.55 ± 7.38 × 10^−2^ mm); the last two did not differ statistically from each other. Gesi et al.^49^, evaluated the CAD of 392 teeth with a single canal (257 TVPNAT and 135 TPNAAPDR), and also confirmed that in the latter, the initial volume of the root canal was significantly greater. As already clarified, permanent dentin production and deposition are the main responsible factors for changing the morphology of the pulp cavity, including the potential constriction of the root canals over time^33–36^. Obviously, both processes are only continuous in vital teeth, as the constant formation of dentin depends on the presence of active odontoblasts. Furthermore, necrotic teeth are often affected by resorptive processes^50^ not always seen in radiographs^51^, which can destroy apical constriction, favoring the occurrence of apexes similar to those of teeth with incomplete rhizogenesis, mimicking “larger CAD”.

Single canals of maxillary central incisors had the largest CAD mean (38.26 ± 6.68 × 10^−2^ mm), while the smallest were observed in the mesiobuccal canals of maxillary (17.00 ± 4.56 x 10^-2^ mm) and mandibular (17.37 ± 4.20 x 10^-2^ mm) first molars, and mesiolingual canals (15.79 ± 3.44 x 10^-2^ mm) of mandibular first molars. The greater volume of maxillary central incisor and canine canals, as well as atresia of the buccal and mesial canals of maxillary and mandibular molars, respectively, have been demonstrated since the classic studies by Hess, in 1925^52^, Pucci and Reig, in 1944^53^, and Pineda and Kuttler, in 1972^54^. However, the techniques available at that time—dye injection, decalcification and clearing of specimens—only allowed visual and subjective analysis. Currently, mathematical measurements can be conducted through imaging exams and softwares^20,21,55,56^. demonstrating, however, similar results to those obtained from the traditional practical method used herein^20,21^.

Kfir et al.^26^, determined in vivo the CAD of 388 canals comparing conventional K-Files and nickel-titanium rotary instruments without taper (Lightspeed) after preparing the cervical and middle thirds using Gates–Glidden drills and K-Files or Profile 04 instruments. The smallest difference between them (6.7 ± 3.0 × 10^−2^ mm) was observed after determining the CAD in single canals of maxillary central incisors, which ranged from 29 to 36 × 10^−2^ mm (approximately)—values slightly lower than those found in this research. Ponce and Fernandez^22^, evaluating 18 maxillary anterior teeth (6 central incisors, 5 lateral incisors and 7 canines), observed that the latter had a wider apical constriction (0.353 mm) than the others (0.298 mm—central incisors; and 0.292 mm—lateral incisors). In addition to the methodological and sampling peculiarities inherent to each study (already mentioned), other factors such as the tactile sense of the clinician and small variations between the real and nominal diameters of the endodontic instruments^20^, can also play a role in the occurrence of different results. In the research by Ponce and Fernandez^22^, for example, all teeth were vital, and had been extracted from patients with a mean age of 42 years, and measurements were performed from histological (two-dimensional) images.

Wolf et al.^55^, carried out an extensive literature review associated with the analysis of the characteristics of apical constriction of 125 mandibular incisors of German patients by means of micro-CT. In teeth with only one canal and one foramen, the anatomical diameter means were 0.24 ± 0.09 and 0.23 ± 0.07 mm for wide and atresic canals, in that order, being, therefore, similar to those reported herein (23.33 ± 6.61 × 10^−2^ mm—mandibular central incisor, and 18.53 ± 4.92 × 10^−2^ mm—mandibular lateral incisor). In a similar study evaluating 109 mandibular first premolars from Swiss and German patients^56^, it was observed that, in teeth that had only one canal and one foramen, the CAD means were 0.37 ± 0.11 mm for wide canals and 0.28 ± 0.09 mm for atresic canals. Similar values were found by Morfis et al.^57^ (0.37 mm), and Wu et al.^58^ (0.35 mm). In the present study, the CAD mean in single canals of mandibular first premolars was 20.83 ± 4.41 × 10^−2^ mm. Similar values were found by Awawdeh et al.^59^ (0.27 ± 0.07 mm—wide canals, and 0.21 ± 0.07 mm—atresic canals), and Arora and Tewari^23^ (0.25 ± 0.12 mm).

Our results demonstrated that the CAD means in single canals of maxillary second premolars and mandibular canines were 27.50 ± 6.38 and 30.00 ± 10.27 × 10^−2^ mm—values respectively similar to those found by Arora and Tewari^23^ (0.24 ± 0.1 mm), and Ponce and Fernandez^22^ (298 µm). In the studies by Wu et al.^60^, and Versiani et al.^61^, the CAD means in the buccolingual and mesiodistal directions in these teeth were 0.47 and 0.36 mm and 0.43 and 0.31 mm, respectively.

Degerness and Bowles^62^, evaluated the anatomical characteristics of maxillary molars under optical microscopy. Similar to the results of the present study, in the vicinities of the apical constriction (0.64 mm from the foramen), the mesiobuccal canals of the maxillary second molars had slightly larger dimensions (0.35 mm in the buccopalatal and 0.28 mm in the mesiodistal directions) than the maxillary first molars (0.29 mm in both directions). However, these index were significantly higher than those found in the present research—17.00 ± 4.56, 18.80 ± 7.67, 18.33 ± 5.00 and 19.44 ± 5.27 × 10^−2^ mm for the mesio and distobuccal canals of maxillary first and second molars, in that order. Almeida et al.^21^, evaluating 108 mandibular molars with independent canals, observed that the CAD means of the mesiobuccal and mesiolingual canals were 0.37 (0.10 ± 0.75 mm) and 0.38 (0.21 ± 0.77 mm), respectively. Similar values were found by Wu et al.^60^, who analyzed under optical microscopy the apical third of horizontally sectioned extracted human teeth, and observed that at 1 mm from the apex, the largest mean diameters were 0.4 (0.2 ± 0.52 mm) for the mesiobuccal and 0.38 (0.32 ± 0.67 mm) for the mesiolingual canals. In the present study, the CAD means in the mesiobuccal and mesiolingual canals of mandibular first and second molars were 19.40 and 17.45 × 10^−2^ mm and 20.79 and 19.99 × 10^−2^ mm, in that order. Regarding these dissimilar results, it should be highlighted that the studies by Degerness and Bowles^62^, Wu et al.^60^, and Almeida et al.^21^ were carried out on extracted teeth and images were used for the determination of the anatomical diameter. Furthermore, in the study by Almeida et al.^21^, only teeth with individual mesial canals (Vertucci type IV) were used. In this research, manual instruments were used for determining the CAD of the root canals, regardless of their classification.

In this scientific investigation, the CAD means of the palatine canals of maxillary first and second molars were 26.80 ± 6.59 and 27.78 ± 7.54 x 10^-2^ mm, respectively. For the distal canals of mandibular first and second molars, these values were 26.32 ± 5.97 and 31.05 ± 9.21 x 10^-2^ mm, in that order. Similar values were reported by Arora and Tewari^23^ (0.32 ± 0.14 and 0.30 ± 0.12 and of 0.30 ± 0.10 and 0.32 ± 0.14 mm).

Since the CAD means of the root canals of different dental groups were significantly influenced by the pulpoperiradicular diagnosis, it would be important to establish them considering individually TVPNAT, TPNNAAPDR and TPNAAPDR. However, in this study, this analysis was not possible due to the small number of root canals/teeth evaluated in certain diagnostic conditions, which is, therefore, its main limitation. Future research with similar methodologies and more robust samples are needed to determine the CAD means of the root canals of teeth affected by different pulpoperiapical diagnosis.

## Conclusions

The values of CAD of the root canals were influenced only by the canal/tooth and pulpoperiradicular diagnosis. The largest CAD was observed in single canals of maxillary central incisors; and the smallest CAD in the mesiobuccal and mesial canals of maxillary and mandibular first molars, respectively. TPNNAAPDR and TPNAAPDR had a significantly larger CAD mean than TPNNAAPDR and TVPNAT, and the last two did not differ statistically from each other.

## Data Availability

The datasets used and/or analysed during the current study is available by the following link: https://1drv.ms/f/s!AnuvHAhxDtNGg-shvI-tPZ0fmL46dw?e=Rx1Vsw.
